# Collaborative models for increasing efficiency of early drug assessment

**DOI:** 10.1186/2052-3211-8-S1-O15

**Published:** 2015-10-05

**Authors:** Anna Nachtnebel, Claudia Wild

**Affiliations:** 1 Ludwig Boltzmann Institute for Health Technology Assessment, Vienna, 1090, Austria

## Introduction

Several countries have set up early awareness and alert systems (EAAS) to inform their customers about technologies that may have a significant impact on the health care system [1].

## Methods

The Austrian Ludwig Boltzmann Institute for Health Technology Assessment (LBI-HTA) implemented an EAAS specifically for oncologic drugs in 2009 [2]. Due to rising costs for cancer therapies, oncologic drugs are of importance throughout Europe and are therefore assessed concurrently by health technology assessment (HTA) agencies leading to considerable redundancies. Experiences of the LBI-HTA with applying different collaborative models used for the reduction of redundancies in the assessment of new oncologic drugs will be described.

## Results

In order to identify potential ways of collaboration a workshop with 12 agencies from nine countries all involved in assessing new oncologic drugs was held in 2010. Following this workshop, the LBI-HTA started to send out "calls for collaboration" to identify partners interested in jointly conducting reports. Up until now, 11 calls have been sent out resulting in 15 collaborations with a total of six institutes. Collaboration initially led to some delays in report production, but since the same agencies repeatedly indicated interest in collaboration, familiarity with processes and development of trust was eventually achieved ultimately leading to efficiency gains.

Besides the active production of joint reports, rapid relative effectiveness assessments produced in international collaboration within the European HTA Network EUnetHTA can serve as basis for local reports [3]. So far, four assessments on pharmaceuticals have been published by EUnetHTA of which one addressed an oncologic drug. This assessment was enriched with local and context-specific information and therefore allowed a fast and less resource-intense production of a report.

## Conslusions

Increasing financial pressure on health care systems and limited research resources necessitate exploring ways of sharing and reusing research findings. Local initiatives driven by individual agencies but also European-wide developments offer the opportunity to produce assessments more efficiently and to reduce redundancies.
